# *N*-Ferrocenylmethyl-2-nitro­aniline

**DOI:** 10.1107/S1600536812039177

**Published:** 2012-09-29

**Authors:** Oumelkheir Rahim, Abdelhamid Khelef, Belgacem Terki, Mohammed Sadok Mahboub, Touhami Lanez

**Affiliations:** a Department of Chemistry, University of Ouargla, PO Box 511, Ouargla 30000, Algeria; b Department of Sciences and Technology, University Mohamed Khider of Biskra, PO Box 145, Biskra 07000, Algeria; c VTRS Laboratory, Institute of Sciences and Technology, University of El-Oued, PO Box 789, El-Oued 39000, Algeria

## Abstract

In the title compound, [Fe(C_5_H_5_)(C_12_H_11_N_2_O_2_)], the two cyclo­penta­dienyl (Cp) rings are nearly eclipsed and parallel to each other, the dihedral angle between their mean planes being 2.54 (1)°. One of the Cp rings is substituted by a nitro­benzenamine group, which is essentially perpendicular to the substituted cyclo­penta­dienyl ring, with an N—C(H_2_)—C—C torsion angle of 89.8 (2)°. Intra­molecular N—H⋯O and N—H⋯N hydrogen bonds occur. In the crystal, weak C—H⋯O hydrogen bonds link adjacent mol­ecules.

## Related literature

For background to the design and properties of ferrocene derivatives, see: Argyropoulos & Coutouli-Argyropoulou (2002 ▶); Cano *et al.* (1995 ▶); Shaabani & Shaghaghi (2010 ▶). For the synthesis of (ferrocenylmeth­yl)trimethyl­ammonium iodide, see: Osgerby & Pauson (1961 ▶). For a related structure, see: Khelef *et al.* (2012 ▶). 
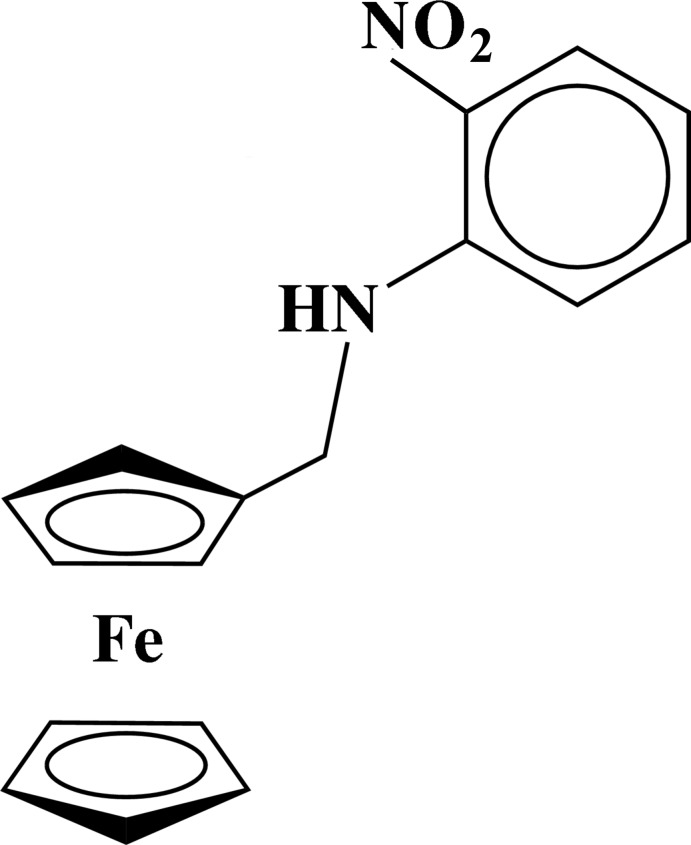


## Experimental

### 

#### Crystal data

[Fe(C_5_H_5_)(C_12_H_11_N_2_O_2_)]
*M* *_r_* = 336.17
Monoclinic, 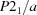

*a* = 10.3609 (3) Å
*b* = 7.8700 (2) Å
*c* = 17.7948 (7) Å
β = 93.043 (2)°
*V* = 1448.95 (8) Å^3^
*Z* = 4
Mo *K*α radiation
μ = 1.05 mm^−1^
*T* = 293 K
0.3 × 0.1 × 0.1 mm

#### Data collection

Nonius KappaCCD diffractometer
14651 measured reflections
3204 independent reflections
2881 reflections with *I* > 2σ(*I*)
*R*_int_ = 0.028

#### Refinement

*R*[*F* ^2^ > 2σ(*F* ^2^)] = 0.025
*wR*(*F* ^2^) = 0.069
*S* = 1.05
3204 reflections
203 parameters
H atoms treated by a mixture of independent and constrained refinement
Δρ_max_ = 0.40 e Å^−3^
Δρ_min_ = −0.25 e Å^−3^

### 

Data collection: *COLLECT* (Nonius, 1998 ▶); cell refinement: *DENZO* and *SCALEPACK* (Otwinowski & Minor, 1997 ▶); data reduction: *DENZO* and *SCALEPACK*; program(s) used to solve structure: *SIR92* (Altomare *et al.*, 1994 ▶); program(s) used to refine structure: *SHELXL97* (Sheldrick, 2008 ▶); molecular graphics: *ORTEP-3* (Farrugia, 1997 ▶); software used to prepare material for publication: *WinGX* (Farrugia, 1999 ▶).

## Supplementary Material

Crystal structure: contains datablock(s) global, I. DOI: 10.1107/S1600536812039177/zq2180sup1.cif

Structure factors: contains datablock(s) I. DOI: 10.1107/S1600536812039177/zq2180Isup2.hkl

Additional supplementary materials:  crystallographic information; 3D view; checkCIF report

## Figures and Tables

**Table 1:** Hydrogen-bond geometry (Å, °)

*D*—H⋯*A*	*D*—H	H⋯*A*	*D*⋯*A*	*D*—H⋯*A*
N1—H10⋯O2	0.827 (16)	2.01 (2)	2.6511 (19)	133.3 (18)
N1—H10⋯N2	0.827 (16)	2.624 (19)	2.961 (2)	106.0 (16)
C4—H4⋯O2^i^	0.93	2.57	3.283 (2)	134

Symmetry code: (i) 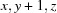.

**Fig. 1.**
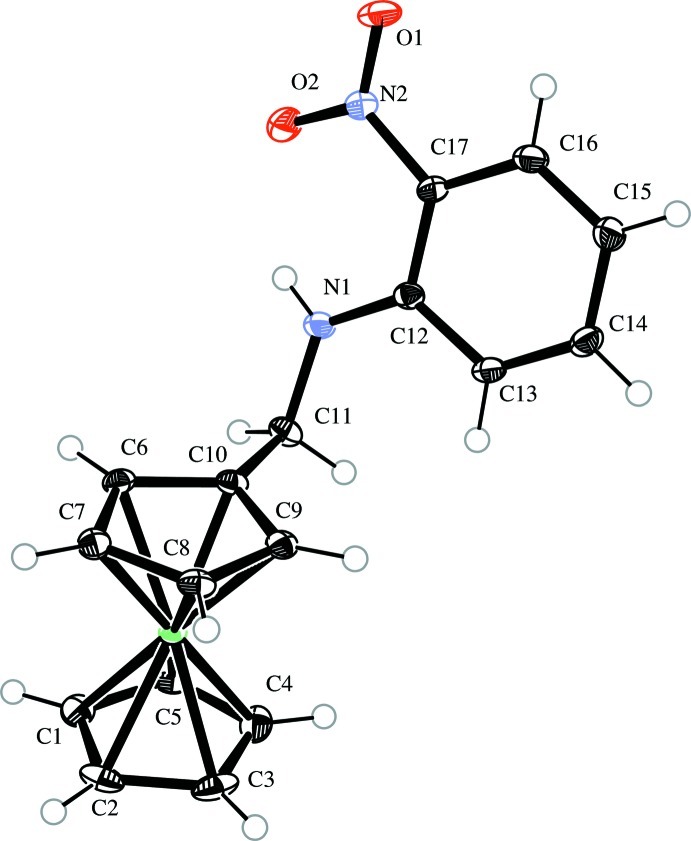
The molecular structure of the title compound, showing the atomic numbering scheme and 50% probability displacement ellipsoids.

## supplementary crystallographic information

### Comment

Ferrocene derivatives have attracted the attention of many groups of researchers because of their utility in organic synthesis (Cano *et al.*, 1995), medicinal chemistry (Argyropoulos & Coutouli-Argyropoulou, 2002) and in electrochemical studies (Shaabani & Shaghaghi, 2010). Herein, as a continuation of our research related to ferrocene derivatives (Khelef *et al.*, 2012), we report the synthesis and X-ray diffraction characterization of the title compound.

In the title compound, [Fe(C_5_H_5_)(C_12_H_11_N_2_O_2_)], the two cyclopentadienyl (Cp) rings are nearly eclipsed and parallel to each other, the dihedral angle between the mean planes is 2.54 (1)°. One of the Cp ring is substituted by a nitrobenzenamine group which is essentially perpendicular to the substituted cyclopentadienyl ring, with a N—C(H_2_)—C—C torsion angle of -79.83 (2)°. Weak C—H···O hydrogen bonds link adjacent molecules (Table 1).

### Experimental

(Ferrocenylmethyl)trimethylammonium iodide was synthesized according to the reported methods of Osgerby & Pauson (Osgerby & Pauson, 1961). *N*-(Ferrocenylmethyl)-2-nitrobenzenamine was synthesized as follows: 2-nitroaniline (2.14 g, 15.48 mmol) was added in small portions to a well stirred solution of (ferrocenylmethyl)trimethylammonium iodide (6 g, 15.48 mmol) in water (120 *cm*^3^). The resulting mixture was then heated at 110–115°C for 6 h. It was then allowed to cool to room temperature. The obtained precipitate was separated by filtration, washed with water to remove any trace of unchanged (ferrocenylmethy)trimethylammonium iodide and finally recrystallized from ethanol 95% to produce the title compound as cinnabar-red needles (4.85 g, 92%; m.p. 110–112°C).

^1^H NMR (CDCl_3_): 4.14 (d, 2H, J = 4.73 Hz, C*H*_2_Fc); 4.21 (t, 2H, J = 1.88 Hz, η^5^-C_5_*H*_4_*ortho*); 4.27 (s, 5H, η^5^-C_5_*H*_5_); 4.28 (d, 2H, η^5^-C_5_*H*_4_*meta*); 6.68 (t, 1H, J = 7.18 Hz, Ar*H*); 6.90 (d, 1H, J = 8.49 Hz, Ar*H*); 7.47 (t, 1H, J = 8.12 Hz, Ar*H*); 8.23 (dd, 1H, J = 8.12 Hz, Ar*H*); 8.37 (s, 1H, N*H*).

^13^C NMR (CDCl_3_): 42.3 (-ve DEPT) (1 C, *C*H2Fc); 67.3 (2 C, η^5^-*C*_5_*H*_4_*meta*); 68.1 (2 C, η^5^-*C*_5_*H*_4_); 68.8 (5 C, η^5^-*C*_5_*H*_5_); 84.5 (1 C, η^5^-*C*_5_*H*_4_); 113.8 (1 C, C_6_H_4_); 115.3 (1 C, *C*_6_H_4_);126.9 (1 C, *C*_6_H_4_); 131.8 (1 C, *C*_6_H_4_); 136.3 (1 C, *C*_6_H_4_); 144.9 (1 C, *C*_6_H_4_).

### Refinement

All hydrogen positions, except H10, were calculated after each cycle of refinement using a riding model, with C—H = 0.93 Å and *U*_iso_(H) = 1.2*U*_eq_(C) for aromatic H atoms, and with C—H = 0.97 Å and *U*_iso_(H) = 1.2*U*_eq_(C) for methylene H atoms. The N-bound hydrogen atom, H10, was located in a difference Fourier map and freely refined.

### Crystal data

**Table:**

[Fe(C_5_H_5_)(C_12_H_11_N_2_O_2_)]	*Z* = 4
*M**_r_* = 336.17	*F*(000) = 696
Monoclinic, *P*2_1_/*a*	*D*_x_ = 1.541 Mg m^−^^3^
Hall symbol: -P 2yab	Mo *K*α radiation, λ = 0.71073 Å
*a* = 10.3609 (3) Å	θ = 1.2–27.4°
*b* = 7.8700 (2) Å	µ = 1.05 mm^−^^1^
*c* = 17.7948 (7) Å	*T* = 293 K
β = 93.043 (2)°	Needle, red
*V* = 1448.95 (8) Å^3^	0.3 × 0.1 × 0.1 mm

### Data collection

**Table:**

Nonius KappaCCD diffractometer	2881 reflections with *I* > 2σ(*I*)
Radiation source: fine-focus sealed tube	*R*_int_ = 0.028
Graphite monochromator	θ_max_ = 27.4°, θ_min_ = 1.2°
Detector resolution: 9 pixels mm^-1^	*h* = −13→13
CCD scans	*k* = −10→10
14651 measured reflections	*l* = −22→22
3204 independent reflections	

### Refinement

**Table:**

Refinement on *F*^2^	Primary atom site location: structure-invariant direct methods
Least-squares matrix: full	Secondary atom site location: difference Fourier map
*R*[*F*^2^ > 2σ(*F*^2^)] = 0.025	Hydrogen site location: inferred from neighbouring sites
*wR*(*F*^2^) = 0.069	H atoms treated by a mixture of independent and constrained refinement
*S* = 1.05	*w* = 1/[σ^2^(*F*_o_^2^) + (0.0381*P*)^2^ + 0.5817*P*] where *P* = (*F*_o_^2^ + 2*F*_c_^2^)/3
3204 reflections	(Δ/σ)_max_ = 0.001
203 parameters	Δρ_max_ = 0.40 e Å^−^^3^
0 restraints	Δρ_min_ = −0.25 e Å^−^^3^

### Special details

**Table:**

Geometry. All e.s.d.'s (except the e.s.d. in the dihedral angle between two l.s. planes) are estimated using the full covariance matrix. The cell e.s.d.'s are taken into account individually in the estimation of e.s.d.'s in distances, angles and torsion angles; correlations between e.s.d.'s in cell parameters are only used when they are defined by crystal symmetry. An approximate (isotropic) treatment of cell e.s.d.'s is used for estimating e.s.d.'s involving l.s. planes.
Refinement. Refinement of *F*^2^ against ALL reflections. The weighted *R*-factor *wR* and goodness of fit *S* are based on *F*^2^, conventional *R*-factors *R* are based on *F*, with *F* set to zero for negative *F*^2^. The threshold expression of *F*^2^ > σ(*F*^2^) is used only for calculating *R*-factors(gt) *etc*. and is not relevant to the choice of reflections for refinement. *R*-factors based on *F*^2^ are statistically about twice as large as those based on *F*, and *R*- factors based on ALL data will be even larger.

### Fractional atomic coordinates and isotropic or equivalent isotropic displacement parameters (Å^2^)

**Table:**

	*x*	*y*	*z*	*U*_iso_*/*U*_eq_	
Fe	0.175327 (16)	0.49618 (2)	0.136596 (10)	0.01155 (8)	
O1	−0.08310 (11)	−0.31659 (13)	0.38282 (7)	0.0288 (3)	
O2	0.09259 (10)	−0.23198 (13)	0.33170 (7)	0.0277 (3)	
N1	0.13730 (11)	0.09690 (15)	0.31440 (7)	0.0173 (2)	
N2	−0.00760 (11)	−0.20171 (15)	0.36602 (7)	0.0190 (2)	
C1	0.34295 (12)	0.55527 (19)	0.08304 (8)	0.0196 (3)	
H1	0.3849	0.4876	0.0491	0.024*	
C2	0.24330 (13)	0.67691 (19)	0.06407 (9)	0.0219 (3)	
H2	0.2089	0.7022	0.0160	0.026*	
C3	0.20652 (13)	0.75263 (18)	0.13377 (10)	0.0244 (3)	
H3	0.1437	0.8360	0.1382	0.029*	
C4	0.28328 (13)	0.67775 (19)	0.19565 (9)	0.0224 (3)	
H4	0.2788	0.7042	0.2464	0.027*	
C5	0.36735 (12)	0.55548 (19)	0.16420 (9)	0.0195 (3)	
H5	0.4272	0.4879	0.1911	0.023*	
C6	0.13517 (12)	0.24086 (17)	0.12877 (8)	0.0155 (3)	
H6	0.1914	0.1558	0.1147	0.019*	
C7	0.04727 (12)	0.33367 (17)	0.07798 (8)	0.0163 (3)	
H7	0.0373	0.3199	0.0261	0.020*	
C8	−0.02175 (12)	0.45125 (18)	0.12293 (8)	0.0162 (3)	
H8	−0.0848	0.5271	0.1049	0.019*	
C9	0.02341 (11)	0.43195 (17)	0.20148 (8)	0.0153 (3)	
H9	−0.0059	0.4929	0.2420	0.018*	
C10	0.12191 (11)	0.30137 (16)	0.20541 (8)	0.0144 (3)	
C11	0.19956 (12)	0.23941 (18)	0.27600 (8)	0.0173 (3)	
H11A	0.2846	0.2037	0.2619	0.021*	
H11B	0.2111	0.3334	0.3110	0.021*	
C12	0.03771 (12)	0.11221 (16)	0.36170 (7)	0.0141 (3)	
C13	−0.00039 (12)	0.27404 (17)	0.38947 (8)	0.0161 (3)	
H13	0.0417	0.3709	0.3735	0.019*	
C14	−0.09834 (13)	0.29185 (17)	0.43954 (8)	0.0181 (3)	
H14	−0.1180	0.3994	0.4573	0.022*	
C15	−0.16826 (13)	0.15031 (18)	0.46392 (8)	0.0201 (3)	
H15	−0.2335	0.1634	0.4974	0.024*	
C16	−0.13745 (14)	−0.00804 (16)	0.43693 (9)	0.0191 (3)	
H16	−0.1841	−0.1025	0.4514	0.023*	
C17	−0.03569 (13)	−0.02881 (17)	0.38750 (8)	0.0157 (3)	
H10	0.1571 (19)	−0.001 (2)	0.3026 (12)	0.028 (5)*	

### Atomic displacement parameters (Å^2^)

**Table:**

	*U*^11^	*U*^22^	*U*^33^	*U*^12^	*U*^13^	*U*^23^
Fe	0.01011 (11)	0.00982 (11)	0.01488 (12)	−0.00088 (6)	0.00230 (8)	0.00070 (6)
O1	0.0366 (6)	0.0115 (5)	0.0392 (7)	−0.0041 (4)	0.0109 (5)	0.0019 (4)
O2	0.0323 (5)	0.0169 (5)	0.0353 (6)	0.0029 (4)	0.0149 (5)	−0.0029 (4)
N1	0.0198 (5)	0.0129 (6)	0.0197 (6)	0.0014 (4)	0.0052 (4)	0.0026 (5)
N2	0.0242 (6)	0.0137 (6)	0.0192 (6)	0.0005 (5)	0.0029 (5)	0.0014 (4)
C1	0.0156 (6)	0.0184 (7)	0.0256 (8)	−0.0041 (5)	0.0079 (5)	−0.0016 (6)
C2	0.0188 (6)	0.0216 (7)	0.0249 (8)	−0.0077 (5)	−0.0011 (5)	0.0106 (6)
C3	0.0159 (6)	0.0108 (6)	0.0470 (10)	−0.0022 (5)	0.0079 (6)	0.0012 (6)
C4	0.0212 (6)	0.0227 (7)	0.0238 (8)	−0.0102 (6)	0.0058 (5)	−0.0074 (6)
C5	0.0113 (5)	0.0196 (7)	0.0274 (8)	−0.0040 (5)	−0.0004 (5)	0.0041 (6)
C6	0.0153 (6)	0.0106 (6)	0.0211 (7)	−0.0020 (5)	0.0037 (5)	0.0001 (5)
C7	0.0154 (6)	0.0164 (6)	0.0172 (7)	−0.0047 (5)	0.0007 (5)	−0.0006 (5)
C8	0.0103 (5)	0.0154 (6)	0.0230 (7)	−0.0019 (5)	0.0012 (5)	0.0034 (5)
C9	0.0125 (5)	0.0144 (6)	0.0196 (7)	−0.0024 (5)	0.0052 (5)	0.0008 (5)
C10	0.0129 (5)	0.0128 (6)	0.0178 (7)	−0.0030 (5)	0.0024 (5)	0.0026 (5)
C11	0.0153 (6)	0.0185 (7)	0.0184 (7)	−0.0019 (5)	0.0027 (5)	0.0039 (5)
C12	0.0159 (6)	0.0129 (6)	0.0132 (6)	0.0004 (5)	−0.0017 (5)	0.0021 (5)
C13	0.0196 (6)	0.0109 (6)	0.0179 (7)	−0.0018 (5)	−0.0004 (5)	0.0017 (5)
C14	0.0227 (6)	0.0124 (6)	0.0189 (7)	0.0032 (5)	−0.0004 (5)	−0.0006 (5)
C15	0.0218 (6)	0.0182 (7)	0.0208 (7)	0.0036 (5)	0.0066 (5)	0.0019 (5)
C16	0.0208 (7)	0.0140 (7)	0.0229 (8)	−0.0007 (5)	0.0052 (6)	0.0039 (5)
C17	0.0197 (6)	0.0104 (6)	0.0169 (7)	0.0015 (5)	0.0014 (5)	0.0016 (5)

### Geometric parameters (Å, º)

**Table:**

Fe—C3	2.0450 (14)	C4—H4	0.9300
Fe—C6	2.0552 (13)	C5—H5	0.9300
Fe—C10	2.0562 (13)	C6—C7	1.4466 (19)
Fe—C9	2.0638 (12)	C6—C10	1.4579 (19)
Fe—C4	2.0669 (14)	C6—H6	0.9300
Fe—C2	2.0691 (14)	C7—C8	1.4382 (19)
Fe—C8	2.0738 (12)	C7—H7	0.9300
Fe—C5	2.0769 (13)	C8—C9	1.458 (2)
Fe—C1	2.0776 (13)	C8—H8	0.9300
Fe—C7	2.0826 (13)	C9—C10	1.4474 (18)
O1—N2	1.2423 (15)	C9—H9	0.9300
O2—N2	1.2549 (15)	C10—C11	1.5348 (18)
N1—C12	1.3711 (17)	C11—H11A	0.9700
N1—C11	1.4790 (17)	C11—H11B	0.9700
N1—H10	0.830 (18)	C12—C13	1.4292 (18)
N2—C17	1.4471 (17)	C12—C17	1.4346 (18)
C1—C2	1.435 (2)	C13—C14	1.3926 (19)
C1—C5	1.453 (2)	C13—H13	0.9300
C1—H1	0.9300	C14—C15	1.4096 (19)
C2—C3	1.445 (2)	C14—H14	0.9300
C2—H2	0.9300	C15—C16	1.3790 (19)
C3—C4	1.449 (2)	C15—H15	0.9300
C3—H3	0.9300	C16—C17	1.4179 (19)
C4—C5	1.432 (2)	C16—H16	0.9300
			
C3—Fe—C6	174.23 (6)	Fe—C3—H3	125.6
C3—Fe—C10	142.98 (6)	C5—C4—C3	107.29 (13)
C6—Fe—C10	41.54 (5)	C5—C4—Fe	70.17 (8)
C3—Fe—C9	112.44 (6)	C3—C4—Fe	68.56 (8)
C6—Fe—C9	68.95 (5)	C5—C4—H4	126.4
C10—Fe—C9	41.13 (5)	C3—C4—H4	126.4
C3—Fe—C4	41.26 (6)	Fe—C4—H4	126.5
C6—Fe—C4	144.34 (6)	C4—C5—C1	108.18 (13)
C10—Fe—C4	111.60 (6)	C4—C5—Fe	69.41 (7)
C9—Fe—C4	107.04 (6)	C1—C5—Fe	69.55 (7)
C3—Fe—C2	41.13 (6)	C4—C5—H5	125.9
C6—Fe—C2	134.76 (6)	C1—C5—H5	125.9
C10—Fe—C2	174.42 (5)	Fe—C5—H5	126.7
C9—Fe—C2	144.32 (6)	C7—C6—C10	109.39 (11)
C4—Fe—C2	69.45 (6)	C7—C6—Fe	70.56 (7)
C3—Fe—C8	108.74 (6)	C10—C6—Fe	69.27 (7)
C6—Fe—C8	68.34 (5)	C7—C6—H6	125.3
C10—Fe—C8	69.41 (5)	C10—C6—H6	125.3
C9—Fe—C8	41.27 (5)	Fe—C6—H6	126.5
C4—Fe—C8	132.87 (6)	C8—C7—C6	107.01 (12)
C2—Fe—C8	114.18 (5)	C8—C7—Fe	69.43 (7)
C3—Fe—C5	68.49 (6)	C6—C7—Fe	68.52 (7)
C6—Fe—C5	115.06 (6)	C8—C7—H7	126.5
C10—Fe—C5	108.15 (5)	C6—C7—H7	126.5
C9—Fe—C5	132.24 (6)	Fe—C7—H7	127.1
C4—Fe—C5	40.42 (6)	C7—C8—C9	108.81 (12)
C2—Fe—C5	68.87 (6)	C7—C8—Fe	70.09 (7)
C8—Fe—C5	172.26 (6)	C9—C8—Fe	69.00 (7)
C3—Fe—C1	68.33 (6)	C7—C8—H8	125.6
C6—Fe—C1	111.03 (5)	C9—C8—H8	125.6
C10—Fe—C1	134.27 (5)	Fe—C8—H8	126.9
C9—Fe—C1	172.94 (6)	C10—C9—C8	108.04 (12)
C4—Fe—C1	68.62 (6)	C10—C9—Fe	69.15 (7)
C2—Fe—C1	40.50 (6)	C8—C9—Fe	69.73 (7)
C8—Fe—C1	145.71 (6)	C10—C9—H9	126.0
C5—Fe—C1	40.94 (6)	C8—C9—H9	126.0
C3—Fe—C7	133.71 (6)	Fe—C9—H9	126.7
C6—Fe—C7	40.92 (5)	C9—C10—C6	106.74 (11)
C10—Fe—C7	69.88 (5)	C9—C10—C11	127.06 (12)
C9—Fe—C7	69.22 (5)	C6—C10—C11	126.19 (11)
C4—Fe—C7	172.91 (5)	C9—C10—Fe	69.71 (7)
C2—Fe—C7	109.79 (6)	C6—C10—Fe	69.19 (7)
C8—Fe—C7	40.49 (5)	C11—C10—Fe	125.33 (8)
C5—Fe—C7	146.44 (6)	N1—C11—C10	113.36 (10)
C1—Fe—C7	115.69 (6)	N1—C11—H11A	108.9
C12—N1—C11	125.30 (12)	C10—C11—H11A	108.9
C12—N1—H10	116.2 (13)	N1—C11—H11B	108.9
C11—N1—H10	118.1 (13)	C10—C11—H11B	108.9
O1—N2—O2	121.78 (12)	H11A—C11—H11B	107.7
O1—N2—C17	118.86 (11)	N1—C12—C13	121.41 (12)
O2—N2—C17	119.36 (11)	N1—C12—C17	123.88 (12)
C2—C1—C5	108.55 (12)	C13—C12—C17	114.71 (11)
C2—C1—Fe	69.43 (7)	C14—C13—C12	122.34 (12)
C5—C1—Fe	69.51 (7)	C14—C13—H13	118.8
C2—C1—H1	125.7	C12—C13—H13	118.8
C5—C1—H1	125.7	C13—C14—C15	121.41 (12)
Fe—C1—H1	126.9	C13—C14—H14	119.3
C1—C2—C3	107.00 (13)	C15—C14—H14	119.3
C1—C2—Fe	70.07 (8)	C16—C15—C14	118.34 (12)
C3—C2—Fe	68.54 (8)	C16—C15—H15	120.8
C1—C2—H2	126.5	C14—C15—H15	120.8
C3—C2—H2	126.5	C15—C16—C17	120.91 (12)
Fe—C2—H2	126.4	C15—C16—H16	119.5
C2—C3—C4	108.99 (12)	C17—C16—H16	119.5
C2—C3—Fe	70.33 (8)	C16—C17—C12	122.25 (12)
C4—C3—Fe	70.18 (8)	C16—C17—N2	116.03 (11)
C2—C3—H3	125.5	C12—C17—N2	121.70 (12)
C4—C3—H3	125.5		
			
C3—Fe—C1—C2	−38.48 (9)	C3—Fe—C7—C8	−64.19 (11)
C6—Fe—C1—C2	135.36 (9)	C6—Fe—C7—C8	118.91 (11)
C10—Fe—C1—C2	177.19 (8)	C10—Fe—C7—C8	81.50 (8)
C4—Fe—C1—C2	−82.98 (10)	C9—Fe—C7—C8	37.47 (8)
C8—Fe—C1—C2	53.30 (14)	C2—Fe—C7—C8	−104.42 (9)
C5—Fe—C1—C2	−120.20 (12)	C5—Fe—C7—C8	174.23 (9)
C7—Fe—C1—C2	90.87 (9)	C1—Fe—C7—C8	−148.06 (8)
C3—Fe—C1—C5	81.72 (9)	C3—Fe—C7—C6	176.90 (8)
C6—Fe—C1—C5	−104.44 (9)	C10—Fe—C7—C6	−37.40 (7)
C10—Fe—C1—C5	−62.61 (11)	C9—Fe—C7—C6	−81.44 (8)
C4—Fe—C1—C5	37.21 (9)	C2—Fe—C7—C6	136.67 (8)
C2—Fe—C1—C5	120.20 (12)	C8—Fe—C7—C6	−118.91 (11)
C8—Fe—C1—C5	173.50 (9)	C5—Fe—C7—C6	55.33 (13)
C7—Fe—C1—C5	−148.93 (8)	C1—Fe—C7—C6	93.04 (8)
C5—C1—C2—C3	0.17 (15)	C6—C7—C8—C9	0.16 (14)
Fe—C1—C2—C3	58.82 (9)	Fe—C7—C8—C9	−58.25 (9)
C5—C1—C2—Fe	−58.65 (9)	C6—C7—C8—Fe	58.42 (8)
C3—Fe—C2—C1	118.47 (12)	C3—Fe—C8—C7	136.60 (9)
C6—Fe—C2—C1	−67.47 (11)	C6—Fe—C8—C7	−38.09 (8)
C9—Fe—C2—C1	171.60 (9)	C10—Fe—C8—C7	−82.77 (8)
C4—Fe—C2—C1	80.77 (9)	C9—Fe—C8—C7	−120.43 (11)
C8—Fe—C2—C1	−150.32 (9)	C4—Fe—C8—C7	176.41 (8)
C5—Fe—C2—C1	37.38 (9)	C2—Fe—C8—C7	92.62 (9)
C7—Fe—C2—C1	−106.74 (9)	C1—Fe—C8—C7	57.81 (13)
C6—Fe—C2—C3	174.06 (8)	C3—Fe—C8—C9	−102.97 (9)
C9—Fe—C2—C3	53.14 (13)	C6—Fe—C8—C9	82.34 (8)
C4—Fe—C2—C3	−37.70 (8)	C10—Fe—C8—C9	37.66 (8)
C8—Fe—C2—C3	91.21 (9)	C4—Fe—C8—C9	−63.16 (11)
C5—Fe—C2—C3	−81.08 (9)	C2—Fe—C8—C9	−146.95 (8)
C1—Fe—C2—C3	−118.47 (12)	C1—Fe—C8—C9	178.24 (9)
C7—Fe—C2—C3	134.79 (8)	C7—Fe—C8—C9	120.43 (11)
C1—C2—C3—C4	−0.05 (15)	C7—C8—C9—C10	0.20 (14)
Fe—C2—C3—C4	59.75 (9)	Fe—C8—C9—C10	−58.72 (8)
C1—C2—C3—Fe	−59.79 (9)	C7—C8—C9—Fe	58.92 (9)
C10—Fe—C3—C2	174.00 (8)	C3—Fe—C9—C10	−147.17 (8)
C9—Fe—C3—C2	−149.68 (8)	C6—Fe—C9—C10	38.87 (8)
C4—Fe—C3—C2	119.75 (11)	C4—Fe—C9—C10	−103.56 (8)
C8—Fe—C3—C2	−105.61 (9)	C2—Fe—C9—C10	178.13 (9)
C5—Fe—C3—C2	82.08 (9)	C8—Fe—C9—C10	119.60 (11)
C1—Fe—C3—C2	37.90 (8)	C5—Fe—C9—C10	−66.42 (10)
C7—Fe—C3—C2	−67.50 (10)	C7—Fe—C9—C10	82.82 (8)
C10—Fe—C3—C4	54.25 (12)	C3—Fe—C9—C8	93.23 (9)
C9—Fe—C3—C4	90.58 (8)	C6—Fe—C9—C8	−80.73 (8)
C2—Fe—C3—C4	−119.75 (11)	C10—Fe—C9—C8	−119.60 (11)
C8—Fe—C3—C4	134.64 (8)	C4—Fe—C9—C8	136.84 (8)
C5—Fe—C3—C4	−37.66 (8)	C2—Fe—C9—C8	58.53 (13)
C1—Fe—C3—C4	−81.85 (9)	C5—Fe—C9—C8	173.98 (8)
C7—Fe—C3—C4	172.75 (7)	C7—Fe—C9—C8	−36.78 (8)
C2—C3—C4—C5	−0.10 (15)	C8—C9—C10—C6	−0.48 (13)
Fe—C3—C4—C5	59.74 (9)	Fe—C9—C10—C6	−59.56 (8)
C2—C3—C4—Fe	−59.84 (9)	C8—C9—C10—C11	178.65 (11)
C3—Fe—C4—C5	−118.75 (12)	Fe—C9—C10—C11	119.57 (12)
C6—Fe—C4—C5	58.96 (13)	C8—C9—C10—Fe	59.08 (9)
C10—Fe—C4—C5	92.96 (9)	C7—C6—C10—C9	0.59 (14)
C9—Fe—C4—C5	136.42 (8)	Fe—C6—C10—C9	59.89 (8)
C2—Fe—C4—C5	−81.17 (9)	C7—C6—C10—C11	−178.55 (11)
C8—Fe—C4—C5	174.41 (8)	Fe—C6—C10—C11	−119.25 (12)
C1—Fe—C4—C5	−37.67 (9)	C7—C6—C10—Fe	−59.30 (9)
C6—Fe—C4—C3	177.71 (8)	C3—Fe—C10—C9	56.33 (12)
C10—Fe—C4—C3	−148.29 (8)	C6—Fe—C10—C9	−117.97 (11)
C9—Fe—C4—C3	−104.83 (8)	C4—Fe—C10—C9	91.48 (8)
C2—Fe—C4—C3	37.58 (8)	C8—Fe—C10—C9	−37.78 (8)
C8—Fe—C4—C3	−66.84 (11)	C5—Fe—C10—C9	134.43 (8)
C5—Fe—C4—C3	118.75 (12)	C1—Fe—C10—C9	172.19 (8)
C1—Fe—C4—C3	81.08 (9)	C7—Fe—C10—C9	−81.10 (8)
C3—C4—C5—C1	0.21 (15)	C3—Fe—C10—C6	174.30 (8)
Fe—C4—C5—C1	58.93 (9)	C9—Fe—C10—C6	117.97 (11)
C3—C4—C5—Fe	−58.72 (9)	C4—Fe—C10—C6	−150.56 (7)
C2—C1—C5—C4	−0.24 (15)	C8—Fe—C10—C6	80.18 (8)
Fe—C1—C5—C4	−58.84 (9)	C5—Fe—C10—C6	−107.60 (8)
C2—C1—C5—Fe	58.60 (9)	C1—Fe—C10—C6	−69.85 (10)
C3—Fe—C5—C4	38.42 (9)	C7—Fe—C10—C6	36.87 (7)
C6—Fe—C5—C4	−146.53 (9)	C3—Fe—C10—C11	−65.38 (14)
C10—Fe—C5—C4	−102.28 (9)	C6—Fe—C10—C11	120.33 (14)
C9—Fe—C5—C4	−62.91 (11)	C9—Fe—C10—C11	−121.71 (14)
C2—Fe—C5—C4	82.72 (9)	C4—Fe—C10—C11	−30.23 (12)
C1—Fe—C5—C4	119.71 (12)	C8—Fe—C10—C11	−159.49 (12)
C7—Fe—C5—C4	176.98 (10)	C5—Fe—C10—C11	12.73 (12)
C3—Fe—C5—C1	−81.29 (9)	C1—Fe—C10—C11	50.48 (14)
C6—Fe—C5—C1	93.75 (9)	C7—Fe—C10—C11	157.20 (12)
C10—Fe—C5—C1	138.00 (8)	C12—N1—C11—C10	−79.74 (16)
C9—Fe—C5—C1	177.38 (8)	C9—C10—C11—N1	89.84 (16)
C4—Fe—C5—C1	−119.71 (12)	C6—C10—C11—N1	−91.19 (15)
C2—Fe—C5—C1	−36.99 (9)	Fe—C10—C11—N1	180.00 (9)
C7—Fe—C5—C1	57.26 (13)	C11—N1—C12—C13	−11.7 (2)
C10—Fe—C6—C7	120.67 (10)	C11—N1—C12—C17	168.50 (13)
C9—Fe—C6—C7	82.17 (8)	N1—C12—C13—C14	−177.79 (13)
C4—Fe—C6—C7	172.30 (8)	C17—C12—C13—C14	1.99 (19)
C2—Fe—C6—C7	−65.41 (10)	C12—C13—C14—C15	−1.9 (2)
C8—Fe—C6—C7	37.70 (8)	C13—C14—C15—C16	0.0 (2)
C5—Fe—C6—C7	−149.87 (8)	C14—C15—C16—C17	1.7 (2)
C1—Fe—C6—C7	−105.40 (8)	C15—C16—C17—C12	−1.6 (2)
C9—Fe—C6—C10	−38.50 (7)	C15—C16—C17—N2	176.83 (13)
C4—Fe—C6—C10	51.63 (12)	N1—C12—C17—C16	179.49 (13)
C2—Fe—C6—C10	173.92 (7)	C13—C12—C17—C16	−0.3 (2)
C8—Fe—C6—C10	−82.97 (8)	N1—C12—C17—N2	1.2 (2)
C5—Fe—C6—C10	89.46 (8)	C13—C12—C17—N2	−178.58 (12)
C1—Fe—C6—C10	133.93 (8)	O1—N2—C17—C16	9.75 (19)
C7—Fe—C6—C10	−120.67 (10)	O2—N2—C17—C16	−169.57 (13)
C10—C6—C7—C8	−0.47 (14)	O1—N2—C17—C12	−171.86 (13)
Fe—C6—C7—C8	−58.99 (9)	O2—N2—C17—C12	8.8 (2)
C10—C6—C7—Fe	58.52 (9)		

### Hydrogen-bond geometry (Å, º)

**Table:**

*D*—H···*A*	*D*—H	H···*A*	*D*···*A*	*D*—H···*A*
N1—H10···O2	0.827 (16)	2.01 (2)	2.6511 (19)	133.3 (18)
N1—H10···N2	0.827 (16)	2.624 (19)	2.961 (2)	106.0 (16)
C4—H4···O2^i^	0.93	2.57	3.283 (2)	134
C16—H16···O1	0.93	2.36	2.683 (2)	100

Symmetry code: (i) *x*, *y*+1, *z*.
